# Comparison of clinical characteristics of patients with pandemic SARS-CoV-2-related and community-acquired pneumonias in Hungary – a pilot historical case-control study

**DOI:** 10.1007/s11357-020-00294-x

**Published:** 2020-11-11

**Authors:** Viktor J. Horváth, Noémi Hajdú, Orsolya Vági, Karolina Schnábel, Emese Szelke, Anna E. Körei, Magdolna Békeffy, Márk M. Svébis, Beatrix A. Domján, Tamás Berényi, István Takács, Zoltán Ungvári, Attila Kun, Ádám G. Tabák

**Affiliations:** 1 Department of Internal Medicine and Oncology, Faculty of Medicine, Semmelweis University, Korányi S. u. 2/a, Budapest, H-1083 Hungary; 2 Emergency Department, Faculty of Medicine, Semmelweis University, Budapest, Hungary; 3 Vascular Cognitive Impairment and Neurodegeneration Program, Reynolds Oklahoma Center on Aging/Oklahoma Center for Geroscience, Department of Biochemistry and Molecular Biology, University of Oklahoma Health Sciences Center, Oklahoma City, OK USA; 4 International Training Program in Geroscience, Doctoral School of Basic and Translational Medicine/Department of Public Health, Semmelweis University, Budapest, Hungary; 5 Department of Health Promotion Sciences, College of Public Health, University of Oklahoma Health Sciences Center, Oklahoma City, OK USA; 6 Department of Obstetrics & Gynaecology, Tolna County Balassa János Hospital, Szekszárd, Hungary; 7 Department of Public Health, Faculty of Medicine, Semmelweis University, Budapest, Hungary; 8 Department of Epidemiology and Public Health, University College London, London, UK

**Keywords:** SARS-CoV-2, Pneumonia, Aging population, Prediction, Case-control study

## Abstract

The distinction between severe acute respiratory syndrome coronavirus 2 (SARS-CoV-2)–related and community-acquired pneumonias poses significant difficulties, as both frequently involve the elderly. This study aimed to predict the risk of SARS-CoV-2-related pneumonia based on clinical characteristics at hospital presentation. Case-control study of all patients admitted for pneumonia at Semmelweis University Emergency Department. Cases (*n* = 30) were patients diagnosed with SARS-CoV-2-related pneumonia (based on polymerase chain reaction test) between 26 March 2020 and 30 April 2020; controls (*n* = 82) were historical pneumonia cases between 1 January 2019 and 30 April 2019. Logistic models were built with SARS-CoV-2 infection as outcome using clinical characteristics at presentation. Patients with SARS-CoV-2-related pneumonia were younger (mean difference, 95% CI: 9.3, 3.2–15.5 years) and had a higher lymphocyte count, lower C-reactive protein, presented more frequently with bilateral infiltrate, less frequently with abdominal pain, diarrhoea, and nausea in age- and sex-adjusted models. A logistic model using age, sex, abdominal pain, C-reactive protein, and the presence of bilateral infiltrate as predictors had an excellent discrimination (AUC 0.88, 95% CI: 0.81–0.96) and calibration (*p* = 0.27–Hosmer-Lemeshow test). The clinical use of our screening prediction model could improve the discrimination of SARS-CoV-2 related from other community-acquired pneumonias and thus help patient triage based on commonly used diagnostic approaches. However, external validation in independent datasets is required before its clinical use.

## Introduction

As the coronavirus disease 2019 (COVID-19) pandemic caused by the severe acute respiratory syndrome coronavirus 2 (SARS-CoV-2) continues to evolve worldwide, an increasing amount of information is becoming available on both its pathophysiology and clinical course including its frequently severe clinical outcome [1]. The mortality associated with COVID-19 is high relative to seasonal influenza infections. Older people and those with underlying chronic conditions may be disproportionately affected by both the disease itself and a more severe course leading to respiratory failure or death [2, 3]. Given the age-related changes of the immune system (immune senescence), it is not surprising that approximately 80% of deaths occurred among patients over the age of 65 and that the most severe outcomes were observed in the very old (age over 85 years) [2, 3]. The chronic conditions that increase the risk of SARS-CoV-2 infection include coronary artery disease, heart failure, cardiac arrhythmias, chronic obstructive pulmonary disease, diabetes mellitus, and current smoking, most of which are also associated with biological ageing [4, 5].

Leading symptoms of COVID-19 include fever, fatigue, cough, extremity pain, and gastrointestinal symptoms [6]. However, these symptoms are frequently blunted in older adults, and, for example, fever response may not correlate with the severity of the disease [7]. After a week or so of mild disease, it may progress, leading to dyspnoea and hypoxaemia and pneumonia, and in severe cases, respiratory failure could develop [8, 9]. As clinical symptoms of community-acquired pneumonia (CAP) and SARS-CoV-2-associated pneumonia are similar in general and although serology-based tests could be quick but have a limited sensitivity for the diagnosis of COVID-19, while the gold standard diagnostic polymerase chain reaction (PCR) tests for SARS-CoV-2 could be time-consuming, a predictive model that uses readily available parameters could improve the discrimination between people with CAP and SARS-CoV-2 pneumonia and thus improve the triage procedure in the emergency department [10, 11].

The present study aims to develop such a prediction tool based on conventional diagnostic findings (medical history, physical examination, basic laboratory data, and chest X-ray) for patients presenting with pneumonia in the emergency department using data of all cases with SARS-CoV-2 pneumonia and a historical control group of CAP cases.

## Methods

### Study design and participants

This is a case-control study of SARS-CoV-2-associated pneumonia cases and historical controls with CAP. Cases were all adult patients (> 18 years of age) admitted with a diagnosis of SARS-CoV-2-associated pneumonia to the Emergency Department of Semmelweis University Faculty of Medicine between 26 March 2020 and 30 April 2020. Controls were adult patients admitted for CAP through the Emergency Department to the 1st Department of Internal Medicine, Semmelweis University Faculty of Medicine between 1 January 2019 and 30 April 2019.

As all people diagnosed with COVID-19 were either transferred to dedicated hospitals or discharged to home quarantine, the outcome of these people was not available for this analysis.

Our institution serves as a secondary referral centre for a suburban area of Budapest, Hungary, with ~ 100 thousand inhabitants. Our institution also serves as a tertiary care centre for haematological patients. As the inclusion of these patients may introduce referral bias, we excluded all patients that were cared for haematological malignancies.

We screened our electronic health record system for admissions with a primary or secondary diagnosis of pneumonia based on the International Statistical Classification of Diseases and Related Health Problems 10th Revision (ICD-10) diagnostic codes of J09-J18. For all cases and controls, we confirmed the diagnosis of pneumonia based on chart review of clinical presentation (for conscious patients, any of fever/chill, dyspnoea, chest pain, or cough/sputum production and unlikely alternative diagnosis, for unconscious patients, unlikely alternative diagnosis) and infiltrate seen on chest x-ray. Some controls with CAP had no infiltrate at admission but their diagnosis was confirmed on repeat x-rays or CT scans. As no follow-up data was available for COVID-19 cases, all patients had to have a positive x-ray at admission.

We performed retrospective chart reviews and collected demographical and medical history, and physical examination data, and laboratory and chest X-ray findings at presentation.

**Fig. 1:**
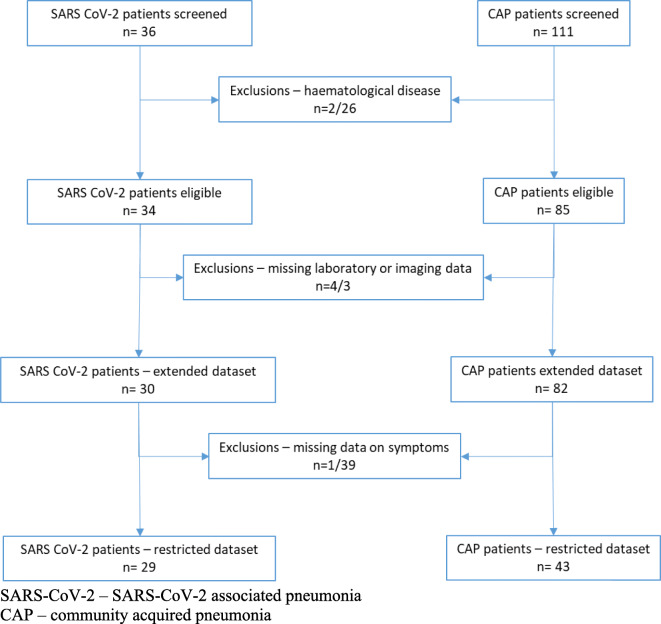
Flow chart of participants. SARS-CoV-2, SARS-CoV-2-associated pneumonia; CAP, community-acquired pneumonia

Of the *n* = 36 patients with COVID-19, we excluded 2 with malignant haematological disease. Of the *n* = 34 eligible patients, *n* = 30 (88.2%) had available laboratory and imaging data and were included in the extended dataset. A further patient had missing data on symptoms, and thus, the restricted dataset includes *n* = 29 (85.3%) patients. For the controls, we excluded *n* = 26 due to haematological disease leaving 85 patients eligible for analysis. We had missing data on laboratory and imaging data for 3 participants; thus, the extended dataset included 82 (96.5%) patients. As data on symptoms were missing for *n* = 39 patients, the restricted dataset only contained 43 (50.6%) patients with data on laboratory data, imaging, and symptoms (Fig. 1).

As no specific study-related procedure was done, no individual consent was required for this retrospective analysis. Ethical approval was obtained from Semmelweis University Regional and Institutional Committee of Science and Research Ethics (RKEB 83/2020).

### Outcome

We can hypothesize that none of the pneumonia cases in early 2019 were related to SARS-CoV-2 and could serve as controls. According to university protocol, all patients with severe respiratory tract infection had a routine screening for SARS-CoV-2 infection using real-time polymerase chain reaction (rtPCR) starting on 13 March 2020 [12].

### Predictors and covariates

*Demographical data* including age and sex were drawn from hospital administrative records. We scanned admission medical history and list of used medications for the following *diseases*: hypertension, diabetes, malignancies, chronic obstructive pulmonary disease (COPD), atrial fibrillation, dementia, and cardiovascular disease (stroke, peripheral arterial disease, or myocardial infarction in medical history).

We recorded the following *physical examination* findings at admission: systolic blood pressure, diastolic blood pressure, heart rate (measured on automated Omron M2 or M4 metres), presence of fever (≥ 37.8 °C).

All *routine laboratory tests* were performed in the same institution (Central Laboratory of Department of Laboratory Medicine, Semmelweis University) on automated systems. Data on blood cell counts, C-reactive protein, and estimated glomerular filtration rate (eGFR–Modification of Diet in Renal Disease equation) were collected.

Among available *radiological investigations*, we selected chest X-rays and (Department of Radiology, Semmelweis University) and reports (by radiology specialists) were screened for the following findings: heart enlargement, decompensation, presence of unilateral or bilateral fluid, and unilateral or bilateral infiltrate.

Unless the patient was unable to provide information, everyone was screened for the following *symptoms*: dyspnoea, chest pain, cough, abdominal pain, diarrhoea, and nausea that were recorded in our dataset.

### Statistical analysis

Descriptive statistics are given as means and standard deviations for continuous and counts and percentages for categorical variables. SARS-CoV-2 and control groups were compared by 2 sample *t* tests and chi^2^ tests as appropriate.

Given the observed large age difference between cases and controls and a potentially increased risk of severe COVID-19 among males [13], age- and sex-adjusted differences were also reported using multiple linear (continuous variables, mean differences, and 95% confidence intervals) and logistic (categorical variables, odds ratios, and 95% confidence intervals) regressions.

Next, we built hierarchical logistic regression models for the prediction of SARS-CoV-2 pneumonia with SARS-CoV-2 status as dependent variable with age and sex entered in the first step; then, further variables with an age- and sex-adjusted association with the outcome (*p* < 0.05) were added in a backward stepwise fashion. The first of these models was run on the extended dataset (*n* = 112) and only included information on laboratory and chest X-ray findings. Then, we built 3 comparable models on the restricted dataset (*n* = 72): using (1) symptoms only, (2) laboratory and chest X-ray findings, and (3) all of these information.

To investigate model discrimination, receiver-operator characteristic (ROC) curves were created and area under the curves (AUC) were calculated with 95% confidence intervals. Model calibration was estimated with the Hosmer-Lemeshow test. To compare the 3 models run on the restricted dataset, we compared the AUCs, calculated incremental discrimination index (IDI), and categorical net reclassification index (NRI) for 2 groups (< 50% and ≥ 50% risk of SARS-CoV-2-associated pneumonia) with model 3 as the reference using STATA [14].

Given the wide age range of our patient population, we also run a sensitivity analysis on the extended dataset after excluding participants younger than 50 years of age.

## Results

### Patient characteristics

Patient characteristics at admission are presented in Table 1. Although SARS-CoV-2 pneumonia cases presented less frequently with hypertension and malignancies and used antiplatelet medications compared to controls, no significant differences between the prevalence of chronic conditions in the medical history, or the use of cardiometabolic medications, were found between the groups after adjustment for age and sex. Similarly, no difference in physical examination findings (blood pressure, heart rate, presence of fever) either in unadjusted or age- and sex-adjusted models were apparent.

**Table 1:** Patient characteristics at admission by SARS-CoV-2 status

	SARS-CoV-2 pneumonia		Historical pneumonia cases			Age- and sex-adjusted models			
	Mean/*n*	SD/%	Mean/*n*	SD/%	*p*	MD/OR	95% CI		*p*
*n*	30		82						
Demographics									
Age (years)	67.2	18.5	74.9	12.6	*0.003*				
Male	20	66.7%	44	53.7%	0.28				
Medical history									
Hypertension	17	56.7%	65	79.3%	*0.029*	0.5	0.19	1.34	0.17
Diabetes mellitus	6	20.0%	26	31.7%	0.25	0.47	0.16	1.38	0.17
Malignancy	2	6.7%	21	25.6%	*0.034*	0.26	0.055	1.21	0.085
Chronic obstructive pulmonary disease	6	20.0%	10	12.2%	0.36	2.04	0.63	6.58	0.24
Atrial fibrillation	4	13.3%	12	14.6%	1	1.19	0.33	4.29	0.79
Dementia	3	10.0%	14	17.1%	0.55	0.74	0.18	2.97	0.67
Cardiovascular disease	6	20.0%	26	31.7%	0.25	0.67	0.23	1.99	0.67
Myocardial infarction	3	10.0%	12	14.6%	0.76	0.69	0.17	2.75	0.59
Stroke	2	6.7%	11	13.4%	0.51	0.57	0.11	2.93	0.50
Peripheral arterial disease	1	3.3%	3	3.7%	1.00	1.58	0.14	17.64	0.71
Cardiometabolic medications									
Antiplatelet medications	10	33.3%	47	57.3%	*0.033*	0.46	0.19	1.16	0.099
Statins	5	16.7%	21	25.6%	0.45	0.58	0.19	1.78	0.34
Angiotensin convertase enzyme inhibitors	9	30.0%	27	32.9%	1	1.18	0.45	3.09	0.74
Angiotensin receptor blockers	1	3.3%	4	4.9%	1	0.43	0.039	4.76	0.49
Beta-blocker	12	40.0%	46	56.1%	0.14	0.68	0.27	1.69	0.41
Calcium channel blockers	7	23.3%	22	26.8%	0.81	1.01	0.36	2.8	0.99
Diuretics	12	40.0%	38	46.3%	0.67	1.18	0.47	3.01	0.72
Metformin	3	10.0%	11	13.4%	0.76	0.66	0.16	2.68	0.56
Sulfonylurea	1	3.3%	3	3.7%	1	1.06	0.10	11.4	0.96
DPP-4 inhibitors	1	3.3%	0	0.0%	0.27	NA			
SGLT2 inhibitors	1	3.3%	1	1.2%	0.47	0.91	0.027	30.8	0.96
GLP-1 receptor agonists	1	3.3%	2	2.4%	1	1.29	0.10	16.3	0.84
Insulin	3	10.0%	12	14.6%	0.76	0.35	0.07	1.71	0.19
Physical examination									
Systolic blood pressure (mmHg)	132	30	128	31	0.62	1.3	− 12.3	14.8	0.85
Diastolic blood pressure (mmHg)	78	14	74	17	0.31	2.7	− 4.7	10.1	0.48
Heart rate (min)	92	15	94	24	0.62	− 4.8	− 14.9	5.3	0.35
Fever	17	56.7%	44	53.7%	0.83	0.93	0.38	2.26	0.87
Laboratory data									
White blood cell count (G/l)	10.9	8.8	13.7	8.6	0.11	− 3.3	− 6.9	0.4	0.078
Neutrophil leukocyte (%)	78.7	10.1	81.8	9.4	0.13	− 3.4	− 7.6	0.9	0.12
Lymphocyte (%)	13.9	8.8	10.5	6.7	*0.033*	3.3	0.1	6.6	*0.043*
Monocytes (%)	6	2.6	6.4	3	0.49	− 0.6	− 1.9	0.7	0.36
C-reactive protein (mg/l)	75.2	59	126.2	89.7	*0.005*	− 52.9	− 89.6	− 16.2	*0.005*
Estimated glomerular filtration rate (ml/min)	69.2	21.8	59.6	28.3	0.066	4.7	− 6.8	16.1	0.42
Imaging data									
Decompensation	10	33.3%	44	53.7%	0.087	0.47	0.19	1.15	0.098
Unilateral fluid collection	7	23.3%	19	23.2%	1	0.94	0.33	2.64	0.90
Bilateral fluid collection	4	13.3%	17	20.7%	0.43	0.97	0.27	3.47	0.96
Unilateral infiltrate	11	36.7%	47	57.3%	0.058	0.42	0.17	1.04	0.061
Bilateral infiltrate	19	63.3%	13	15.9%	*< 0.0001*	9.00	3.34	24.27	*< 0.0001*
Symptoms									
*n*	29		43						
Dyspnoea	14	48.3%	18	41.9%	0.64	1.41	0.53	3.75	0.50
Chest pain	6	20.7%	7	16.3%	0.76	1.05	0.29	3.87	0.94
Cough/sputum production	11	37.9%	22	51.2%	0.34	0.47	0.17	1.32	0.15
Abdominal pain	1	3.4%	10	23.3%	*0.041*	0.069	0.008	0.62	*0.017*
Diarrhoea	0	0.0%	8	18.6%	*0.018*	-	-	-	-
Nausea	1	3.4%	10	23.3%	*0.041*	0.12	0.013	0.998	*0.0499*
Number of pneumonia defining symptoms					0.79				
0	5	17.2%	8	18.6%		1.08	0.29	4.02	0.90
1	5	17.2%	10	23.3%		0.69	0.20	2.34	0.55
> 1	19	65.5%	25	58.1%		1.23	0.44	3.43	0.70

Italics refer to *p* < 0.05

Pneumonia defining symptoms: fever/chill, dyspnoea, chest pain, or cough/sputum production

Mean and standard deviation (SD) for continuous and *n* % for categorical variables

*p* values for unadjusted differences are from 2-sample *t* tests and chi^2^ tests as appropriate

Mean differences (MD) and 95% confidence intervals (95% CI) for continuous and odds ratios (OR) and 95% CIs for categorical variables in age- and sex-adjusted models

Regarding laboratory data, we found a significantly higher proportion of lymphocytes among white blood cells and a substantially lower CRP level in cases with SARS-CoV-2-associated pneumonia that was robust for age and sex adjustment (mean difference [MD]: 3.3, 95% CI: 0.1–6.6%, − 53, 95% CI: 16–90 mg/l, respectively) (Table 1).

Among the investigated imaging findings, we found a much higher proportion of bilateral infiltrate among SARS-CoV-2 cases even after age and sex adjustment (odds ratio [OR]: 9.0, 95% CI: 3.3–24.7) (Table 1).

Although data on presenting symptoms was available only on a limited number of patients, it is notable that control patients with CAP had a substantially higher risk of reporting abdominal symptoms, such as abdominal pain, diarrhoea, and nausea probably partly related to the older age and altogether more frequent comorbidities of the control group.

### Logistic models for the prediction of SARS-CoV-2-associated pneumonia

**Fig. 2:**
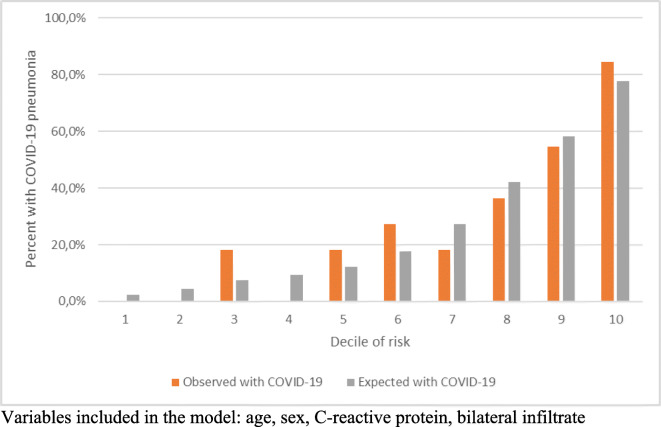
Observed and expected risk of SARS-CoV-2-associated pneumonia in deciles of expected risk according the model developed on the extended dataset. Variables included in the model: age, sex, C-reactive protein, bilateral infiltrate

According to the logistic regression model using laboratory and chest X-ray findings on the extended dataset, we found that younger age, lower CRP, and the presence of bilateral infiltrate are independent predictors of SARS-CoV-2-associated pneumonia and that the model has an excellent discrimination with an ROC-AUC of 0.85 (95% CI: 0.77–0.93). This model also has good calibration according to the Hosmer-Lemeshow test that is graphically represented by the calibration plot. We found good agreement between observed and predicted risks with predicted risk ranging from less than 10% to almost 80%. Our sensitivity analysis after excluding younger patients confirmed results from the main analysis (data available on request) (Table 2, Fig. 2).

**Table 2:** Independent predictors of SARS-CoV-2 pneumonia and model performance characteristics

	Extended dataset		Restricted dataset					
			Model 1		Model 2		Model 3	
	OR	95% CI	OR	95% CI	OR	95% CI	OR	95% CI
Age (years)	0.97	0.93–1.00	0.97	0.93–1.01	0.99	0.95–1.03	0.98	0.93–1.03
Male sex	1.33	0.44–4.01	2.78	0.93–8.27	1.88	0.53–6.72	2.93	0.76–11.40
Abdominal pain	-	-	0.06	0.006–0.546	-	-	0.06	0.005–0.66
C-reactive protein (mg/l)	0.99	0.98–0.998	-	-	0.99	0.98–0.998	0.99	0.98–0.997
Bilateral infiltrate	9.82	3.42–28.20	-	-	14.29	3.80–53.70	12.06	2.95–49.29
AUC	0.85	0.77–0.93	0.74	0.62–0.85	0.84	0.75–0.93	0.88	0.81–0.96
Hosmer-Lemeshow goodness of fit	5.83	*p* = 0.67	4.69	*p* = 0.79	8.1	*p* = 0.42	9.88	*p* = 0.27

Other variables available for the models: lymphocyte percentage (extended dataset), nausea, vomiting (model 1), lymphocyte percentage (model 2)

**Fig. 3:**
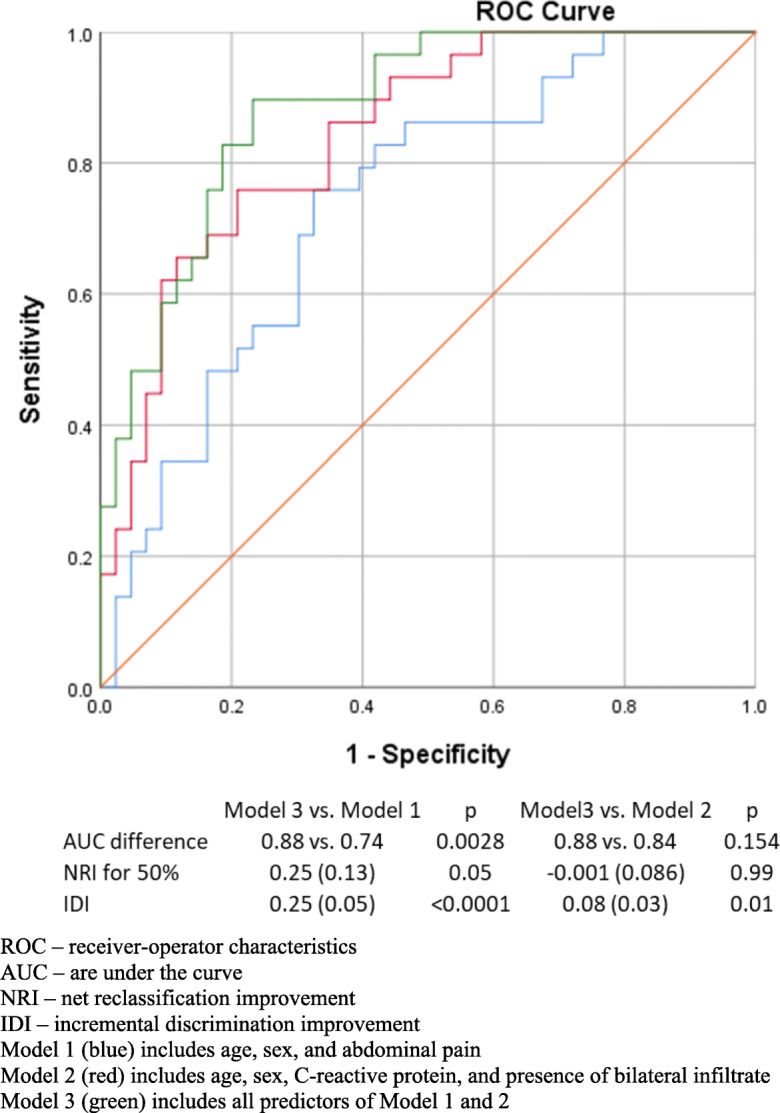
ROC curves and model performance characteristics for models developed on the restricted dataset for the prediction of SARS-CoV-2-associated pneumonia. ROC, receiver-operator characteristics; AUC, area under the curve; NRI, net reclassification improvement; IDI, incremental discrimination improvement. Model 1 (blue) includes age, sex, and abdominal pain. Model 2 (red) includes age, sex, C-reactive protein, and presence of bilateral infiltrate. Model 3 (green) includes all predictors of models 1 and 2

All 3 models run on the restricted dataset had good calibration based on the Hosmer-Lemeshow test (Table 2). When the model based on symptoms and that based on laboratory and chest X-ray findings were compared to the reference model including all potential predictors, we found that the symptom-based model performed significantly worse than the full model both in terms of discrimination (ROC-AUC) and reclassification (NRI and IDI). The model based on laboratory and chest x-ray findings performed similarly to the full model in terms of discrimination (ROC-AUC) and reclassification (NRI), although the IDI of the full model was significantly (by 8%) better compared to model 2 (Fig. 3).

## Discussion

As both SARS-CoV-2-associated and community-acquired pneumonias present frequently in older people (those older than 65) but their contagiousness and treatment are different, the discrimination of these entities is a high public health priority [15, 16]. In the present case-control study with historical controls, we found that routinely collected information at the emergency department could well discriminate (with ROC AUCs above 80%) between pneumonias associated with SARS-CoV-2 and other community-acquired pneumonias. There was an increased risk of SARS-CoV-2-associated pneumonia in younger patients with lower CRP levels, with bilateral infiltrate on chest x-ray and in the absence of abdominal pain. A model even without information on symptoms performed similarly in terms of discrimination and reclassification compared to the full model, suggesting that this model could be used even in unconscious patients. Furthermore, the model showed good calibration with estimated risks between 10 and almost 80%.

Although community-acquired pneumonia affects all ages (i.e. 0.5–1% of the adult population is diagnosed in the UK every year), the risk of acquiring the disease, or being hospitalized with the disease, is higher in older ages [17, 18]. Furthermore, more than half of pneumonia-related deaths occur in very old people (older than 84 years) [17]. The incidence of community-acquired pneumonia and hospitalization due to CAP is highest in adults 65 to 79 years old, and even higher in those with a known viral cause [16, 19, 20]. In contrast, the mean age of patients with SARS-CoV-2-associated pneumonia is much younger (50 to 60 years) [21, 22]. Even patients who required intensive care had a mean age around 65 years [23, 24]. These observations however are not inconsistent with our findings as SARS-CoV-2-associated cases were almost 8 years younger compared to historical controls. It should be noted that our hospitalized cases were substantially older than previously reported cases, probably reflecting the case mix of our institute [21, 22]. While these observations seem to be counterintuitive given the notion that COVID-19 is an ageing disease, it should be emphasized that risk factors of acquiring the disease and that of severity or mortality could be rather different: while the mean age of patients presenting with SARS-CoV-2-associated pneumonia is approximately 67 years in our sample, 93% of deceased patients were older than 60 years according to Hungarian data from the same time period [25].

Similarly, the fact that none of the chronic diseases seemed to be associated with an elevated risk of SARS-CoV-2-related pneumonia could be explained by the increased prevalence of chronic diseases compared in the control group of our study (patients with community-acquired pneumonia) [26].

The fact that no difference in the use of different medications was found in our sample is in line with the recently reported null-findings related to different groups of antihypertensive medications including inhibitors of the renin-agiotensin-aldosterone system [27].

Fever, crackles on auscultation, hypoxemia, and tachycardia considered to be typical signs of radiologically confirmed pneumonia. Altogether these signs and symptoms have a positive predictive value of 57.1% for community-acquired pneumonia [28]. The differentiation between viral and bacterial causes of pneumonia is not possible based on symptoms and physical findings in general, although patients with viral pneumonia were older and more frequently frailer patients with more comorbidities. The clinical observation that viral aetiologies are less likely to cause sputum production, and if present, tends to be watery or scant, while bacterial aetiologies more frequently associated with mucopurulent sputum are not substantiated [29]. In line with the literature, we found no significant difference between the presence of airway symptoms between patients with and without SARS-CoV-2-associated pneumonia. There are however two observations that are worth noting here. First, SARS-CoV-2-associated pneumonia cases in our series were younger than CAP cases, and thus, age might help the differentiation of SARS-CoV-2 cases from other viral pneumonias. Second, we found that gastrointestinal symptoms were less frequently reported by SARS-CoV-2-associated pneumonia cases even after adjustment for other potential predictors. Although this finding may be interesting within our hospital, we suspect that this finding probably relates to the older age and comorbidity/frailty status of CAP cases that our adjustment could not fully capture, or may reflect case mix–specific bias.

While the laboratory findings in SARS-CoV-2-associated pneumonia are unequivocal with generally normal leukocyte count and lymphopenia [8, 21, 22], the findings in community-acquired pneumonia may reflect the aetiology, with abnormal white blood cell counts (high or low) and relative lymphopenia in bacterial cases and mostly normal findings in viral cases [29]. Similar to these observations, we found slightly elevated white blood cell counts in SARS-CoV-2 cases with lymphopenia, while controls had similar white blood cell count but a lower proportion of lymphocytes. C-Reactive protein is a good marker of inflammation, and it is not surprising that it is elevated in both SARS-CoV-2-associated pneumonia and control pneumonia cases. The significantly lower level observed in our SARS-CoV-2 cases (75 mg/l) well corresponds to reported values of approximately 50 mg/l in other clinical cohorts [21, 22]. The higher observed CRP values in the historical pneumonia cases probably relates to the fact that a substantial proportion of community-acquired pneumonias are caused by a bacterial pathogen [16, 18].

Findings from chest x-rays in SARS-CoV-2-associated pneumonias are similar to other viral aetiologies with bilateral infiltrate being the most frequent finding [8, 21, 22, 29]. Given this observation, it is not that surprising how well the presence of a bilateral infiltrate discriminates SARS-CoV-2 associated from community-acquired pneumonias in our study. However, it should be emphasized that none of the thoracic imaging methods, symptoms, or routine laboratory findings, nor their combination (as used in the present prediction algorithm) are diagnostic for SARS-CoV-2-related pneumonia by themselves.

The question arises whether the use of chest computed tomography (CT) instead of chest X-ray would improve the prediction of SARS-CoV-2-related pneumonia. While CT has a higher sensitivity compared to chest X-ray, still 20–25% of CT images will be unremarkable at presentation in people who later develop pneumonia [30, 31]. Due to the fact that most of our patients had no CT images (probably reflecting usual care in the emergency department), we were unable to compare prediction using different imaging methods.

The main novelty of our report is the finding that using easily accessible information (age, C-reactive protein level, presence of bilateral infiltrate, and abdominal pain), we could separate people with SARS-CoV-2-associated pneumonia from those with other aetiologies. We suspect that the good performance of this prediction model relates to the fact that patients present to the emergency department with more severe cases with an advanced disease that already shows the full clinical picture. We also think that the case mix of patients could have a substantial effect on the performance of this model, and thus, external validation is essential before its clinical use.

Our analysis has some weaknesses that have to be acknowledged. First of all, the low number of included patients limited the statistical power of this study. Fortunately, the number of known COVID-19 cases remained relatively low in Hungary, probably owing to the timely introduction of social distancing interventions [32]. Indeed, only 60 new patients were diagnosed with SARS-CoV-2 infection after our database was finalized at our institution until the end of May (Tamás Berényi, personal communication). Given this, we could detect only large effect sizes and some potentially important risk factors could not be investigated. Our control group included community-acquired pneumonia cases of divergent aetiologies, with no known pathogen in most of the cases. This weakness makes our analysis prone to bias related to the case mix of the control group. While this is an important limitation, we think that our model could work in our hospital and could also have external validity in institutions with a similar case mix to ours. Furthermore, the aetiology of community-acquired pneumonia remains unknown in most cases in clinical practice [18, 33]. Our study is lacking external validation, so the findings probably reflect overly optimistic model performance. As historical controls were used in our study, systematic changes in departmental protocols (such as those associated with the emergence of COVID-19) could have led to information bias. While these could affect information on symptoms and chest x-ray reports, it is unlikely for other potential predictors.

The strengths of our study include its population-based nature, the low number of missing cases for laboratory and radiology results, and use of the gold standard method for the diagnosis of SARS-CoV-2 infection. Furthermore, laboratory and radiological examinations were performed in the same institution for both cases and controls. As the number of COVID-19 cases remained limited in Hungary, a case-control design is the optimal setting where characteristics of this disease can be investigated.

In conclusion, we found that some routinely collected data, such as age, C-reactive protein, and the presence of bilateral infiltrate on chest X-ray, can well differentiate patients presenting with pneumonia into groups with high and low risk of SARS-CoV-2 infection. This information can be used in the triage and placement of these patients while the result of the definitive diagnostic test becomes available. Before these findings could be used in clinical practice, they require further validation in larger, independent datasets.

## Data Availability

The datasets and/or code generated during the current study are available from the corresponding author upon reasonable request.
